# The Role of the Interactions at the Tungsten Disulphide Surface in the Stability and Enhanced Thermal Properties of Nanofluids with Application in Solar Thermal Energy

**DOI:** 10.3390/nano10050970

**Published:** 2020-05-18

**Authors:** Paloma Martínez-Merino, Antonio Sánchez-Coronilla, Rodrigo Alcántara, Elisa I. Martín, Iván Carrillo-Berdugo, Roberto Gómez-Villarejo, Javier Navas

**Affiliations:** 1 Departamento de Química Física, Facultad de Ciencias, Universidad de Cádiz, E-11510 Puerto Real (Cádiz), Spain; paloma.martinez@uca.es (P.M.-M.); rodrigo.alcantara@uca.es (R.A.); ivan.carrillo@uca.es (I.C.-B.); roberto.gomezvi@uca.es (R.G.-V.); 2 Departamento de Química Física, Facultad de Farmacia, Universidad de Sevilla, E-41012 Sevilla, Spain; 3 Departamento de Ingeniería Química, Facultad de Química, Universidad de Sevilla, E-41012 Sevilla, Spain; elisamf@us.es

**Keywords:** nanofluids, heat transfer, concentrating solar power, thermal conductivity, tungsten disulphide

## Abstract

Transition metal dichalcogenides (TMCs) exhibit unique properties that make them of interest for catalysis, sensing or energy storage applications. However, few studies have been performed into nanofluids based on TMCs for heat transfer applications. In this study, nanofluids based on 2D-WS_2_ are prepared by liquid phase exfoliation to analyze their potential usage in concentrating solar power plants. Periodic-Density Functional Theory (DFT) calculations were performed to rationalize the success of the exfoliation process. The hydrogen bond interaction between the hydroxyl group from PEG, which acts as a surfactant, and the S atoms of the WS_2_ surface stabilizes the nanosheets in the fluid. Electron localization function (ELF) analysis is indicative of the stability of the S–H interaction from WS_2_ with the molecules of surfactant due to the tendency to interact through weak intermolecular forces of van der Waals solids. Moreover, improvements in thermal properties were also found. Isobaric specific heat increased by up to 10% and thermal conductivity improved by up to 37.3%. The high stability of the nanofluids and the thermal improvements were associated with the high surface area of WS_2_ nanosheets. These results suggest that these nanofluids could be a promising heat transfer fluid in concentrating solar power plants.

## 1. Introduction

The depletion of fossil fuel resources and global warming are two of the most worrying problems in our society. Roughly 70% of all anthropogenic Greenhouse Gas (GHS) emissions derive from the energy sector, with the largest contribution made by CO_2_ from fossil fuel combustion [1]. Furthermore, according to predictions, oil production rates will peak in 2025 at 120 Mbpd but will decline to 40 Mbpd by 2115 because of the oil depletion produced by the high consumption [2]. In this scenario, the use of renewable energy sources is considered to be one of the key solutions to the growing energy demand. Concentrating solar power (CSP) is a type of clean, renewable energy with a global technical potential of almost 3 × 10^6^ TWh/year and which is capable of reducing GHG emissions by an average of 1 kg for each kW generated [3,4]. Decreasing the high cost of this kind of energy to make it economically comparable to fossil fuels is an interesting milestone to reach. Consequently, new types of materials, collector designs, thermal energy storage systems and electrical conversion of CSP plants are being researched [5,6,7,8,9]. In this sense, one of the main research lines that has emerged in recent years is based on replacing the heat transfer fluid (HTF) used in concentrating solar power plants with nanofluids [10,11]. Nanofluids are colloidal suspensions of nanometric solid particles dispersed within a fluid. Choi first introduced the term nanofluid in 1995 and reported enhancements in the thermal properties of nanofluids compared to the original fluids without nanoparticles [12]. Since then, several studies have analyzed the improvements in isobaric specific heat and thermal conductivity of these systems and their possible applications in fields such as electronics, biomedicine, nuclear reactor technology, power generation and others [13,14,15,16,17,18,19,20]. Regarding CSP, nanoparticles not only have a remarkable effect on improving thermal transfer and conductivity of the original heat transfer fluid, but nanoparticles also increase the absorption of incident solar radiation, leading to improvements in the global efficiency of solar plants [21,22]. Sokhansefat [23] reported a theoretical study about the use of Al_2_O_3_/synthetic oil nanofluid in a parabolic trough collector and concluded an average increase of convection heat transfer coefficient by about 14% with a 5% nanoparticle volumetric concentration. Heris [24] and Rehan [25] observed experimentally that Al_2_O_3_/water nanofluids present a higher thermal efficiency than CuO/water and Fe_2_O_3_/water nanofluid, respectively. In a numerical study, Mwesigye [26] found a maximum thermal enhancement of 13.9% with Ag/Therminol VP-1 nanofluid while 12.5% and 7.2% enhancements were obtained for Cu/Therminol VP-1 and Al_2_O_3_/Therminol VP-1. Furthermore, because of the increase in solar irradiation absorption caused by nanoparticles, some authors have proposed the use of nanofluid-based direct solar collectors. Kasaeian [27] studied MWCNT/ethylene glycol and nanosilica/ethylene glycol nanofluids as working fluids in a direct absorption parabolic trough collector. The best results were obtained for the 0.3% MWCNT/EG nanofluid, with an optical efficiency of 71.4% and a thermal efficiency enhancement of 17%. Menbari [28] also found thermal enhancements of up to 52% in direct absorption parabolic trough collectors with the use of CuO/water nanofluids.

For example, there is a necessity to develop more experimental works about nanofluids based on the eutectic mixture of biphenyl and diphenyl oxide commonly used in CSP which are not as researched as water-based nanofluids. In addition, nanoparticles sedimentation is another important issue that requires a great attention for researchers. In this work, nanofluids based on WS_2_ nanosheets dispersed in a eutectic mixture of biphenyl and diphenyl oxide are studied since the high aspect ratio of the two-dimensional nanostructures confers a great stability on nanofluids and improves the heat transfer process. Nanosheets of WS_2_ are created inside the thermal oil through liquid phase exfoliation (LPE), a process in which the inter-layer van der Waals forces of bulk WS_2_ crystal are broken by ultrasound in a liquid medium. Some factors of the process such as surfactant concentration, time and frequency of ultrasound were analyzed to find the best condition to obtain stable WS_2_ nanofluids. Polyethylene glycol is the surfactant used to reduce the potential energy barrier of the exfoliation process and prevents nanosheet agglomeration. In this sense, there is a lack of research in the literature about the interactions between surfactant, solid and liquid medium during the LPE process. Nevertheless, in this work, periodic Density Functional Theory (DFT) calculation and electron localization function (ELF) were performed to advance in the interaction knowledge of the molecules involved in the WS_2_ exfoliation and justify the suitability of polyethylene glycol as surfactant in the exfoliation process. Finally, the stability and thermal properties of the WS_2_ nanofluids have been studied to analyze their viability as working fluids in CSP plants.

## 2. Materials and Methods

### 2.1. Nanofluids Preparation

Four nanofluids based on WS_2_ nanosheets were prepared by liquid phase exfoliation (LPE) from WS_2_ bulk material. In this methodology, 0.015 g of WS_2_ (nanopowder 90 nm, purity ≈ 99%, Sigma-Aldrich, St Louis, MO, USA) and 5 mL of the base fluid were added to four vials to prepare 20 mL of nanofluid. The base fluid is a eutectic mixture of biphenyl (C_12_H_10_, 16.5%) and diphenyl oxide (C_12_H_10_O, 73.5%) supplied by The Dow Chemical Company (Midland, MI, USA) with polyethylene glycol (PEG, molecular weight ≈ 400, Sigma-Aldrich) used as the surfactant. The eutectic mixture of biphenyl and diphenyl oxide is the heat transfer fluid (HTF) commonly used in CSP plants. A methodology previously designed in our laboratory was used to determine the appropriate concentration of PEG in the HTF [29]. The PEG concentrations used help to make the ratio of surface tension components of the fluid similar to that of the WS_2_, leading to a favorable thermodynamic situation for the dispersion of the nanostructures in the fluid. The PEG concentrations used are 0.20 wt. % PEG in the HTF and 0.75 wt. % PEG in the HTF. The lower concentration fits the ratio of surface tension components well as is shown in [29]. The higher concentration is also tested for evaluating if higher concentrations lead to new effects. The mixture of WS_2_ and the base fluid was placed in an ultrasonic bath (Elmasonic P30H, 80 kHz, or Elmasonic TI-H5, 130 kHz) for 4 or 8 h. The temperature was controlled between 25–30 °C to avoid the agglomeration process of nanostructures during the exfoliation. After sonication, the resulting colloidal suspensions were centrifuged twice in a Digicen 20-R centrifuge, Orto Alresa. The first centrifugation was 10 min at 1000 rpm to remove the unexfoliated WS_2_ flakes. Subsequently, the supernatant liquid was subjected to a second centrifugation at 4000 rpm for 10 min for elimination of the aggregated nanomaterial. Finally, the liquid obtained in the second centrifugation is the nanofluid based on the 2D nanostructures of WS_2_. Following this process, four nanofluids have been prepared which differ in the concentration of surfactant and in the time and frequency of ultrasound. Table 1 shows the conditions under which the WS_2_ nanofluids were prepared.

### 2.2. Characterization of Nanofluids

Morphological characterization was performed by transmission electron microscopy (TEM) in a JEM-2100F microscope supplied by Jeol (Akishima, Japan), to verify the formation of 2D nanostructures and determine their nominal size. Since the stability of a nanofluid has a marked influence in its thermal properties, several techniques were used to analyze the dispersion of nanostructures in the HTF. Thus, UV-Vis spectroscopy makes it possible to study the sedimentation process by evaluating the changes in the extinction coefficient over time. UV-Vis spectra were recorded between 400 and 800 nm using an Ocean Optics DH-2000-Bal halogen lamp and an Ocean Optics USB-2000+ monochromator (Amersham, UK). The extinction coefficient was evaluated at *λ* = 629 nm, where there is a characteristic band of WS_2_ [30]. The particle size was measured by dynamic light scattering (DLS), a technique for analyzing the agglomeration process of colloidal suspensions. In turn, *ζ* potential measurements were performed to analyze the electric potential between the medium and the stationary layer attached to nanostructures, which provides information about repulsive forces between nanostructures. Particle size and *ζ* potential measurements were performed using a Zetasizer Nano ZS system supplied by Malvern Instruments Ltd. (Malvern, UK). The stability of WS_2_ nanofluids was evaluated for 30 days using the aforementioned techniques.

Finally, the heat transfer enhancements were calculated as a function of several properties of the nanofluids, such as density, dynamic viscosity, isobaric specific heat and thermal conductivity. Density was measured by pycnometry while dynamic viscosity was determined using a vibrational viscometer, model SV-10 supplied by Malvern Instruments Ltd. (Malvern, UK). Moreover, isobaric specific heat measurements were performed using a temperature modulated differential scanning calorimeter (TMDSC), model DSC 214 Polyma, supplied by Netzsch (Selb, Germany). To perform these measurements, a program was created which has been previously reported [29]. Furthermore, thermal conductivity values for the nanofluids were obtained indirectly from the thermal diffusivity measurements obtained by the laser flash technique (LFA 1600 equipment, supplied by Linseis Thermal Analysis, Selb, Germany). The relationship between thermal conductivity and thermal diffusivity is given by the equation [31,32,33] $k\left(T\right)=D\left(T\right)\cdot C_{P}\left(T\right)\cdot \rho \;\left(T\right)$ where *k* is the thermal conductivity, *D* is the thermal diffusivity, *C*_P_ is the isobaric specific heat and *ρ* is the density (ASTM E14-61-01).

### 2.3. Computational Details

Vienna Ab Initio Simulation Package (VASP) [34,35,36,37] was used for performing periodic density functional theory calculations with the projector-augmented wave method [38,39]. The methodology used in this paper was described previously [29] and we used it for the study of WS_2_ monolayer, (P63/mmc space group in the bulk phase) [40]. Vaspview [41] and Chemcraft 1.6 [42] were the software used for describing electron localization function (ELF) [43,44,45,46,47] pictures and structure, respectively.

## 3. Results and Discussions

**Figure 1:**
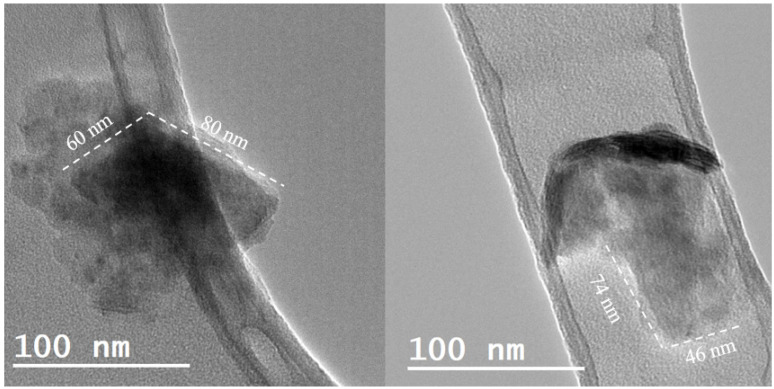
Transmission electron microscopy (TEM) images of the WS_2_ nanosheets found in nanofluids prepared by liquid phase exfoliation (LPE).

TEM analysis was performed to study the size and morphology of the nanostructures obtained by the LPE process. The WS_2_ nanostructures found in all the nanofluids were nanosheets with lateral dimensions between 45–80 nm, as seen in Figure 1. The high dispersion and electrotransparency of the nanosheets are evidence of a successful exfoliation process.

**Figure 2:**
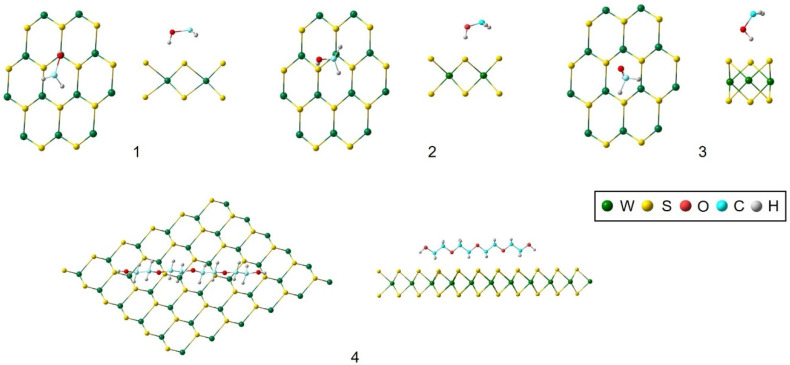
Local geometry for the interaction of polyethylene glycol (PEG) with the WS_2_ surface.

The success of the exfoliation process can be understood from the analysis of the interaction between WS_2_ nanosheets, the surfactant and the molecules of the fluid, studied from periodic-DFT calculations. Thus, the interaction sites of the PEG with the WS_2_ nanosheet were studied based on their interaction energies. The interaction sites are shown in Figure 2. For the interaction with the surface, four binding sites were selected. For the selection of such sites it was defined as the reference point the H of the H–O group of the PEG over a W, the S atoms and over the gap between three S and W, structures **1**, **2** and **3** in Figure 2, respectively. The interaction in parallel with the H atoms over the surface of the WS_2_ nanosheet was also analyzed (**4** in Figure 2). For clarity purposes, only the terminal region of the surfactant molecule that interacts with the surface of the WS_2_ has been shown in Figure 2 for the images of structures **1**–**3**.

The interaction energies (*E*_int_) associated with the binding sites described above (Figure 2) are included in Table 2. This *E*_int_ is defined as

(1) $$E_{int}=E_{\left(WS_{2}+PEG\right)}-E_{\left(WS_{2}\right)}-E_{\left(PEG\right)}$$

where $E_{\left(WS_{2}+PEG\right)}$, $E_{\left(WS_{2}\right)}$ and $E_{\left(PEG\right)}$ are the total energies of the PEG with the WS_2_ monolayer (001) surface, the bare (001) surface of WS_2_ and the PEG, respectively. Based on the interaction energy, the most stable interaction of PEG surfactant with the WS_2_ surface involves structure **2**, that corresponds with the binding site directly over the S (**2**) followed by the interaction on top of W (**1**) and the gap (**3**), the interaction in parallel over the WS_2_ monolayer (**4**) being the least favored. Thus, it is understandable that structure **2** is favored due to the direct hydrogen bonding interaction between the S atom from the WS_2_ and the H atoms from PEG molecule. With the energy considerations, structure **2** will be selected for the discussion from now on.

**Figure 3:**
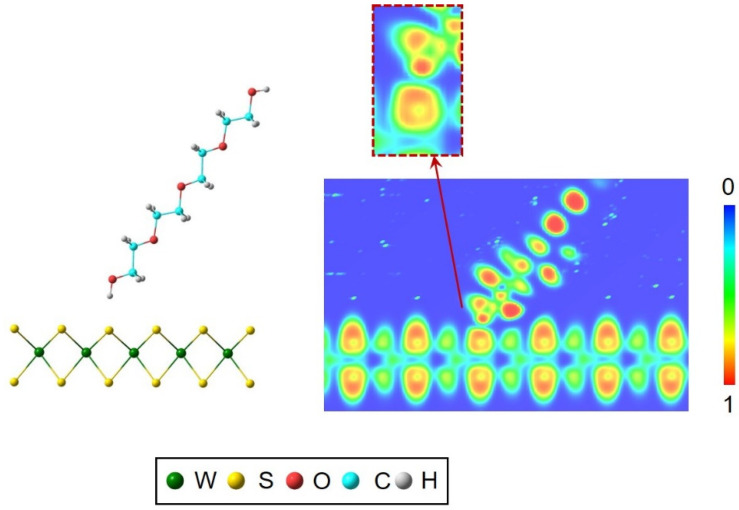
Electron localization function (ELF) contour plots for structure **2**.

The electronic properties around the interaction site in structure **2** will be discussed by analyzing the electron localization function. ELF provides an interesting depiction of the bonding based on the probability of finding electrons around neighbor elements ranging from 0 to 1 [43,44,45,46,47]. The ELF plots focused on the H atom of the H–O group from PEG molecule are shown in Figure 3. The colors of the plots are indicative of the probability of finding electrons in a region of the space between the H from PEG and S from WS_2_. A modification of the contour plot around the O–H–(S_sheet_) interaction can be observed. The directionality of this interaction is shown in the dotted square of the enlarged image in Figure 3. The overlap shown by the green-blue color electron localization outlines between S and the H from the surfactant is indicative of the stability of the S–H interaction. This stability is understandable in van der Waals solids such as WS_2_ because they present a certain tendency to interact with other molecules through weak intermolecular forces [29]. So, the ELF results may provide information of interest for giving feedback in the preparation of the nanofluid. According to the ELF results, nanosheet WS_2_ structures may be favored with the presence of hydrogen bonding surfactants such as PEG. In the present case, the ELF analysis corroborates the hydrogen bond interaction between the hydroxyl terminal group from the PEG molecule and the S atoms from the surface of the WS_2_, which stabilizes the nanosheet when the exfoliation process is applied.

**Figure 4:**
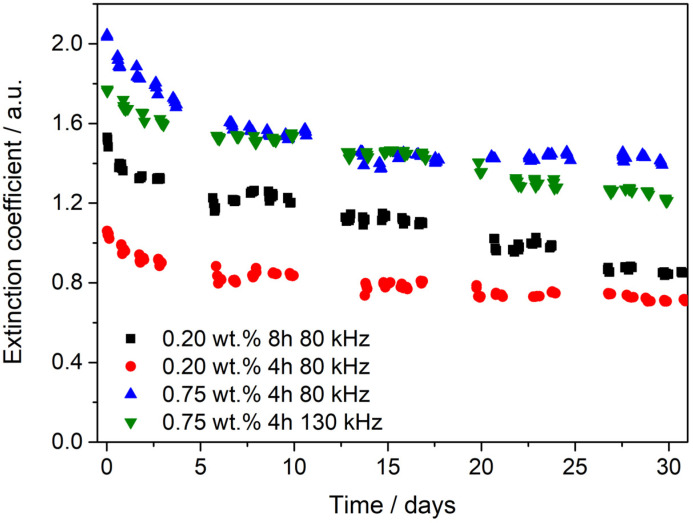
Evolution of the extinction coefficient values at *λ* = 629 nm from UV-Vis spectra over time.

Several measurements were performed involving different techniques to assess the stability of the nanofluids since agglomeration and sedimentation processes lead to negative effects on heat transfer, such as pressure drops and even clogging of the pipes through which nanofluids circulate. Accordingly, UV-Vis spectra, particle size and zeta potential measurements were analyzed for thirty days. Figure 4 illustrates the evolution over time of the extinction coefficient obtained at *λ* = 629 nm from UV-Vis spectra for the nanofluids. At that value of wavelength there is a characteristic absorption band of WS_2_ [30]. Initially, the highest extinction coefficient values were found for the nanofluids prepared with 0.75 wt. % of PEG in HTF, which reveals that the exfoliation process is more successful using this concentration of PEG. Among the nanofluids prepared with 0.75 wt. % of PEG, the nanofluid prepared using a sonication frequency of 80 kHz seems to be more stable since the extinction coefficient remains invariable after 13 days while the nanofluid prepared using a frequency of 130 kHz began to be stable in the 21st day. In the case of the nanofluids prepared with 0.20 wt. % of PEG in HTF, the initial extinction coefficient values were lower than those of the nanofluids prepared with the higher PEG concentration, which means that less nanomaterial was obtained in the exfoliation process. Among these, the nanofluid prepared by means of an LPE process for 4 h seems to be more stable than the one prepared during 8 h, in which case the decrease of the extinction coefficient with time is about 45%. This nanofluid is the one that shows the least stability over time, so increasing the sonication time does not result in increased stability. After 30 days, the nanofluids with the same amount of PEG showed similar extinction coefficient values, which means that the concentration of PEG has a greater influence than the sonication time and frequency on the amount of nanomaterial in the nanofluid during the LPE process. Moreover, although the extinction coefficient of 2D-WS_2_-based nanofluids decreases during the first days, the values after characterization are still high, which is of great interest for solar application since not only could the thermal properties of the HTF be improved but also the absorption of solar radiation may increase. Therefore, the most interesting nanofluid in terms of stability seems to be that prepared using a concentration of PEG of 0.75 wt. % and a sonication frequency of 80 kHz for 4 h because it shows the highest extinction coefficient value after 30 days being stable.

**Figure 5:**
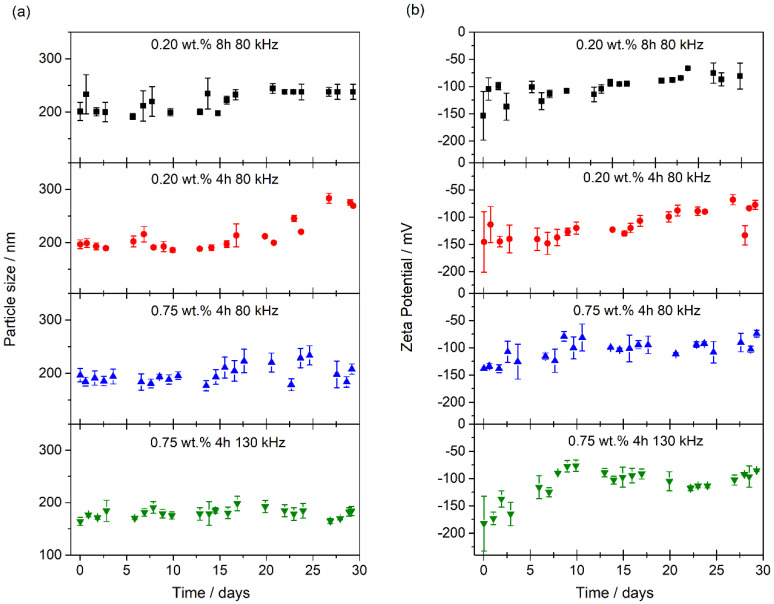
Evolution with time of particle size (**a**) and *ζ* potential (**b**) for the nanofluids prepared.

The evolution of the extinction coefficient is strongly dependent on the agglomeration of nanostructures since the fast sedimentation of clusters reduces the extinction coefficient values. Figure 5a shows the particle size measured by the DLS technique. The particle sizes for all the nanofluids were found to be between 150–250 nm. Nanofluids based on WS_2_ nanosheets show high stability since no significant changes in particle size were observed over time. However, we can observe that after 30 days the nanofluids prepared using a PEG concentration of 0.20 wt. % presented higher values (240 nm for 80 kHz for 8 h and 270 nm for 80 kHz for 4 h) than those obtained for the nanofluids prepared using 0.75 wt. % of PEG, which showed values of 200 nm in the case of the one prepared using 80 kHz and 180 nm for the one using 130 kHz. Probably, these values are not high enough to produce a strong sedimentation in the nanofluids. Therefore, the higher the concentration of PEG, the smaller the particle size.

The particle size analysis was consistent with the *ζ* potential measurements, which reveal the repulsive forces between nanostructures. Accordingly, high absolute values of *ζ* potential are associated with electrically stabilized colloids. However, when the *ζ* potential value is low, there are no repulsive forces between the nanostructures and they tend to agglomerate. It is usually observed that *ζ* potential values near to ±45 mV are predominant in stable nanofluids but when the *ζ* potential is above ±60 mV the stability is excellent [48]. Figure 5b shows the *ζ* potential measurements of nanofluids over time. In the first days after the preparation of the nanofluids, the *ζ* potential measurements showed great variability due to changes in the colloidal systems but the values became stable after the tenth day. The *ζ* potential values of all the nanofluids prepared were close to −100 mV, which indicates that nanofluids present excellent stability over time, as was observed in the UV-Vis and particle size analyzes.

The incorporation of nanostructures into a base fluid is known to increase the density and viscosity of the fluid. The analysis of these properties is vitally important since they affect nanofluid efficiency. An increase in density generally leads to enhanced thermal properties when there is no significant sedimentation. Nevertheless, highly viscous nanofluids have the disadvantage of increasing the pumping power required and producing pressure drop problems [49,50]. Table 3 shows the density and viscosity values of the nanofluids studied and HTF. The density of the HTF at 25 °C was measured obtaining a value of 1056.6 ± 0.5 kg·m^−3^, which is consistent with the value provided by the supplier (1055.7 kg·m^−3^). The density measurements of the nanofluids were similar to that of the HTF, the largest increase of 1.5% seen in the nanofluid prepared with 0.20 wt.% of PEG and applying 80 kHz for 4 h. Both nanofluids prepared using the lowest concentration of PEG show the highest density values and these nanofluids presented higher particle size values, as is described above.

In addition, volume fractions (*φ*) of the nanofluids were calculated from density values, according to $\varphi =\left(\rho_{nf}-\rho_{bf}\right)/\left(\rho_{2D-WS2}-\rho_{bf}\right)$, where *ρ* is the density, and the subscripts *nf* and *bf* are related to the nanofluid and the base fluid, respectively. The values obtained for the volume fraction are shown in Table 3. Higher values are found for nanofluids prepared with the lower PEG concentration, which also showed higher particle size. This means particle size has an important role in volume fraction values found. Moreover, the dynamic viscosity of the HTF was also measured and the result was 3.70 ± 0.02 mPa·s at 25 °C, which is similar to the value provided by the supplier (3.71 mPa·s). No significant viscosity changes are appreciated in the 2D-WS_2_ nanofluids compared to HTF, increases of up to 2% being found. Therefore, these nanofluids would not be expected to present problems of sedimentation or clogging in the pipes through which they circulate in solar plants.

Density and dynamic viscosity are also used to study the performance of the nanofluids by means of the heat transfer coefficient (*h*), which can be expressed as $h=\left(k^{a}\rho^{b}C_{P}^{c}/\mu^{d}\sigma^{e}\right)$ where *k* is the thermal conductivity, *ρ* is the density, *C*_P_ the isobaric specific heat, *µ* the dynamic viscosity and *σ* the surface tension [51,52]. The superscripts a, b, c, d, and e are empirical or theoretical constants that depend on different boundary and geometrical conditions. Furthermore, the constant *e* is normally zero for conventions without phase change [53].

**Figure 6:**
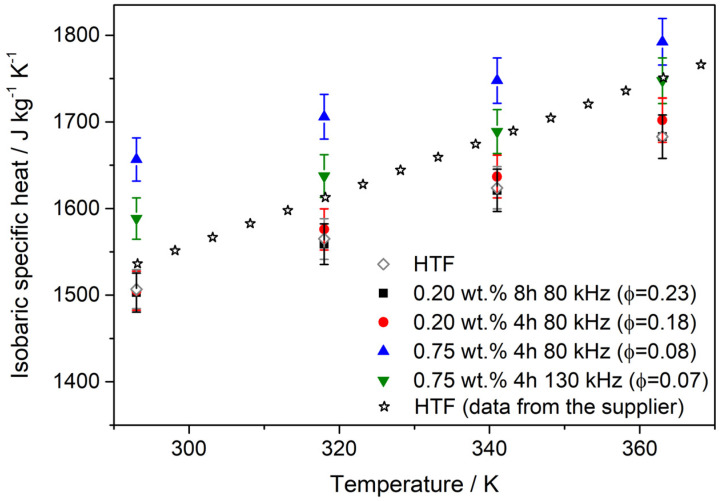
Isobaric specific heat values for nanofluids and pure HTF. The values for pure HTF from the supplier are included for comparison purposes.

The isobaric specific heat was measured by TMDSC. Figure 6 shows the values measured, and also the values for the pure HTF provided from the supplier for comparison purposes. The values measured were coherent with these ones. Additionally, the values measured for the base fluids (HTF + PEG) were quite similar to the values measured for the pure HTF. So, we analyzed the values with respect to the pure HTF because it is the fluid typically used in CSP plants, and in this way, we can compare it with the fluid used now. Contrary to what the classical models claim, some WS_2_ nanofluids show improvements in isobaric specific heat compared to the base fluid. According to these theories, nanofluids should have a lower isobaric specific heat than the base fluid since solids present lower isobaric specific heat than liquids [54,55,56]. However, some researchers have observed the opposite behavior. The increase in the isobaric specific heat in nanofluids can be understood if nanofluids are not considered a mixture of two components but instead take into account the surface interactions between the surfactant molecules, the liquid and the nanostructures [57,58].

Several studies indicate that isobaric specific heat enhancement in nanofluids is associated with the increase in the interfacial thermal resistance produced by the formation of a solid-like thin liquid layer between nanostructures and liquid [59,60,61,62]. In the present work, WS_2_ nanofluids showed higher values than the HTF, and the largest values were found in nanofluids with the highest PEG concentration, that is with lower volume fraction, which means there is an optimum concentration for isobaric specific heat enhancements. This is an exceptional behavior, which has been reported previously [63,64]. Probably, this occurs for an optimum configuration of the surfactant-nanomaterial pair, which leads to a high interfacial thermal resistance value. The isobaric specific heat increased by up to 10% for the nanofluid based on 2D-WS_2_ prepared with 0.75 wt. % of PEG and applying 80 kHz for 4 h, which has volume fraction of 0.08 vol. %. This increase is referred to the values measured for the pure HTF because they were measured in the same conditions in our lab. Additionally, the enhancement in isobaric specific heat found in this work is interesting because, to our knowledge, the higher increase found in nanofluids based in TMCs is about 7.6% for MoSe_2_-based nanofluids [65].

**Figure 7:**
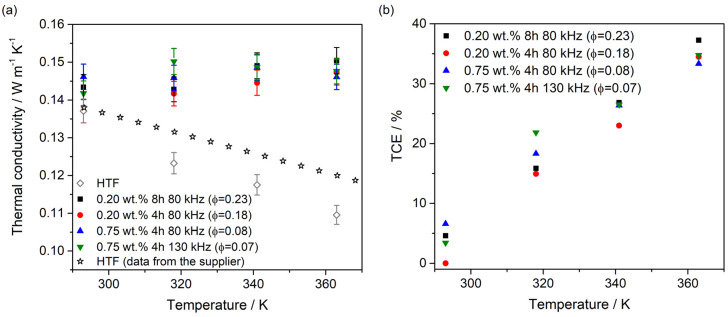
Thermal conductivity for nanofluids and pure HTF (**a**) and percentages of thermal conductivity enhancements for nanofluids compared to HTF (**b**). The values for pure HTF from the supplier are included for comparison purposes.

In turn, thermal conductivity measurements showed the most important enhancements. Figure 7a illustrates the thermal conductivity results for the nanofluids and HTF evaluated between 290 K and 363 K. The values for the pure HTF provided from the supplier are also shown for comparison purposes. The values measured were coherent with these ones. Furthermore, the values measured for the base fluids (HTF + PEG) were quite similar to the values measured for the pure HTF. So, we analyzed the values with respect to the pure HTF because it is the fluid typically used in CSP plants, and in this way, we can compare with the fluid used now. An increasing trend, contrary to that obtained for the HTF, is observed with the temperature in the thermal conductivity values for the 2D-WS_2_. This is probably due to the transport mechanisms in the nanofluids being different to that produced in the fluid. Thus, thermal conductivity enhancement (TCE) was estimated for the nanofluids prepared according to $TCE\left(\%\right)=\left[\left(k_{nf}-k_{bf}\right)/k_{bf}\right]$ [66,67,68], where *k_nf_* is the thermal conductivity for the nanofluids and *k_bf_* is the thermal conductivity for the base fluid. The values obtained of TCE are shown in Figure 7b. The enhancement of thermal conductivity obtained for the 2D-WS_2_-based nanofluids was up to 37.3% with regard to the base fluid, which is particularly interesting for solar applications. The values of thermal conductivity do not show a clear tendency with nanomaterial concentration as is usually observed for water-based nanofluids. In this case, the base fluid is different and also the volume fraction of the nanofluids is very small in comparison with those typically reported. The increase observed is referred to the values measured for the pure HTF because they were measured in the same conditions in our lab. This enhancement is similar to that reported for WS_2_-based nanofluids using CTAB as surfactant, which means the surfactant can help to reach a certain stability, but, in this kind of nanofluid does not affect the thermal conductivity significantly [69].

Moreover, from the results obtained for the nanofluids prepared, it is possible to evaluate their efficiency in heat transfer processes in CSP applications. To this end, the heat transfer coefficient of the base fluid and of the nanofluids was estimated. For this, we used the Nusselt number, which is defined from Reynolds and Prandtl numbers as [70,71]

(2) $$Nu=0.023Re^{0.8}Pr^{0.4}$$

where the Reynolds number is defined as

(3) $$Re=\frac{\rho V_{av}D_{i}}{\mu }$$

where *ρ* is the density, *V_av_* is the mean flow rate, *D_i_* the inner diameter of the pipe and *μ* is the viscosity. The Prandtl number is defined as

(4) $$Pr=\frac{\mu C_{P}}{k}$$

where *C*_P_ is the isobaric specific heat and *k* is the thermal conductivity. Nu is also related with the heat transfer coefficient, *h*, from the equation [72]

(5) $$Nu=\frac{hD_{i}}{k}$$

**Figure 8:**
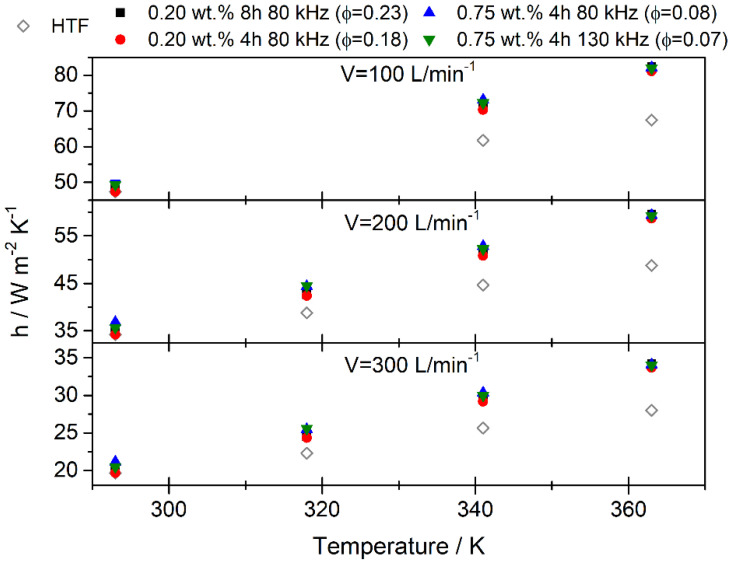
Heat transfer coefficients for 2D-WS_2_ nanofluids and HTF for three flow rates, 100, 200 and 300 L/min.

Considering these equations, we can find the exponents in the expression of heat transfer coefficient for density, viscosity, thermal conductivity and isobaric specific heat as was defined previously, being 0.8, −0.4, 0.6 and 0.4, respectively. Therefore, heat transfer coefficient was estimated for the pure heat transfer fluid and for the nanofluids at three flow rates, 100, 200 and 300 L/min. The values obtained for the heat transfer coefficients are shown in Figure 8. The results show an increase in the heat transfer coefficient when the flow rate increases, as expected. Additionally, the results show an enhancement in the heat transfer processes when nanofluids are used. Furthermore, this enhancement increases with temperature. The 2D-WS_2_ nanofluids in this study were prepared with different PEG concentrations and ultrasonic conditions but heat transfer is seen to improve in all cases. The nanofluid with the highest volume fraction (prepared using a PEG concentration of 0.20 wt. % and sonication frequency of 80 kHz for 8 h) is the nanofluid which shows the highest increase, up to 22.1%. Furthermore, the nanofluids with the lowest concentration of PEG were less stable and present less nanomaterial in the base fluid so it is likely that the presence of PEG is not beneficial in terms of thermal properties but it helps the fluids to reach temporal stability. For the nanofluid with a volume fraction of 0.08 vol. % (prepared using a PEG concentration of 0.75 wt. % and a sonication frequency of 80 kHz for 4 h), an increase in the heat transfer coefficient of 21.7% was found. This nanofluid was the most stable and the changes in the improvement of efficiency (with respect to the maximum obtained, 22.1%) is of the order of the uncertainty of the measurements, so this nanofluid would appear to be the most promising in terms of stability and thermal properties.

## 4. Conclusions

Nanofluids based on WS_2_ nanosheets were prepared and characterized to determine their likely performance in concentrating solar power plants. Several parameters, such as surfactant concentration, sonication frequency and time, were analyzed to improve the liquid phase exfoliation process. Periodic-DFT calculations were performed to rationalize the success of the exfoliation process. The hydrogen bond interaction between the hydroxyl group from PEG, which acts as a surfactant, and the S atoms of the WS_2_ surface stabilizes the nanosheets within the fluid. ELF analysis is indicative of the stability of the S–H interaction, which is understandable in van der Waals solids such as WS_2_ because of their tendency to interact through weak intermolecular forces. The stability analysis showed no significant changes in particle size and *ζ* potential, which was reflected in low sedimentation levels observed by means of UV-Vis. The least stable nanofluid was the one prepared with the longer sonication time. Therefore, increasing the sonication time does not enhance the stability of the nanofluids prepared. However, the increase in the concentration of PEG resulted in stability improvements.

In addition, the viscosity of the nanofluids was similar to that of the base fluid, a slight increase in dynamic viscosity values being observed. These results are advantageous for the use of nanofluids in solar power plants since they would not cause the problems of obstruction and pressure drops characteristic of viscous liquids. Thermal properties were also analyzed. The isobaric specific heat increased for the nanofluids studied by up to 10% while thermal conductivity improved by up to 37.3% with regard to the HTF. It was observed that heat transfer increased in all the nanofluids by up to 22.1% even though they were prepared using different PEG concentrations and sonication conditions. According to these results, the typical eutectic mixture of diphenyl oxide and biphenyl has been optimized by the addition of WS_2_ nanosheets. Therefore, the nanofluids prepared in this work are a promising alternative for use in concentrating solar power plants.

## Figures and Tables

**Table 1:** Conditions used for preparing nanofluids.

Nanofluid	wt. % PEG	Sonication Time/h	Sonication Frequency/kHz
1	0.20	8	80
2	0.20	4	80
3	0.75	4	80
4	0.75	4	130

**Table 2:** Interaction energies associated to the binding sites from Figure 2.

Position	Figure 2	*E*_int_/eV
Over W	**1**	−0.233
Over S	**2**	−0.242
Over the gap	**3**	−0.103
Parallel	**4**	3.224

**Table 3:** Density, volume fraction and viscosity values of heat transfer fluid (HTF) and 2D-WS_2_-based nanofluids at 25 °C.

Sample	Density/kg·m^−3^	*φ*/vol. %	Viscosity/mPa·s
HTF	1056.6 ± 0.5	-	3.70 ± 0.02
HTF + 0.20 wt. % PEG	1057.4 ± 0.4	-	3.71 ± 0.02
HTF + 0.20 wt. % PEG	1057.7 ± 0.6	-	3.71 ± 0.02
0.20 wt. % 8 h 80 kHz	1072.1 ± 1.2	0.23	3.73 ± 0.02
0.20 wt. % 4 h 80 kHz	1069.3 ± 0.7	0.18	3.77 ± 0.01
0.75 wt. % 4 h 80 kHz	1062.8 ± 0.2	0.08	3.76 ± 0.02
0.75 wt. % 4 h 130 kHz	1062.6 ± 0.2	0.07	3.74 ± 0.02

## Abbreviations

**Nomenclature**		
*C*_P_	Isobaric specific heat (J·(kg^−1^·K^−1^))
*D*	Thermal diffusivity (m^2^·s^−1^)
*D*_i_	Inner diameter of the pipe (m)
*E*_int_	Interaction energy (eV)
*E*_PEG_	Energy of PEG molecule (eV)
*E*_WS2_	Energy of the bare surface of WS_2_ (eV)
*E*_WS2+PEG_	Energy interaction of the PEG with the (001) WS_2_ monolayer (eV)
*h*	Heat transfer coefficient (W·(m^−2^·K^−1^))
*k*	Thermal conductivity (W·(m^−1^·K^−1^))
*Nu*	Nusselt number
*Pr*	Prandtl number
*Re*	Reynold number
TCE	Thermal conductivity enhancement (%)
*V*_av_	Mean flow rate (m·s^−1^)
*μ*	Dynamic viscosity (Pa s)
*ρ*	Density (kg·m^−3^)
*λ*	Wavelength (m)
**Subscripts**		
bf	Base fluid
nf	Nanofluid
**Abbreviations**		
CSP	Concentrating solar power
DFT	Density functional theory
DLS	Dynamic light scattering
ELF	Electron localization function
HTF	Heat transfer fluid
LFA	Laser flash analysis
LPE	Liquid phase exfoliation
PEG	Polyethylene glycol
TEM	Transmission electron microscopy
TMDC	Transition metal dichalcogenide
TMDSC	Temperature modulated differential scanning calorimeter
VASP	Vienna Ab Initio Simulation Package
